# Psychological Distress in COPD Assessed by DASS-21-R: Multivariable Regression and Bayesian Analysis Across GOLD Stages

**DOI:** 10.3390/medsci14010147

**Published:** 2026-03-19

**Authors:** Adina Deliu, Luana Alexandrescu, Bogdan Cimpineanu, Oana Cristina Arghir, Sanda Jurja, Ioan Tiberiu Tofolean, Rodica Gabriela Enache, Ioana Gherghisan, Ionela Preotesoiu, Ionut Valentin Stanciu, Andreea Nelson Twakor, Monica Cordos, Alexandra Herlo, Daria Maria Alexandrescu, Doina Ecaterina Tofolean

**Affiliations:** 1 Doctoral School, Faculty of General Medicine, “Ovidius” University, 900470 Constanta, Romania; adina.deliu@365.univ-ovidius.ro (A.D.); oana.arghir@365.univ-ovidius.ro (O.C.A.); sanda.jurja@365.univ-ovidius.ro (S.J.); ioana.husaru@gmail.com (I.G.); ionela.preotesoiu@365.univ-ovidius.ro (I.P.); ionutstvalentin@gmail.com (I.V.S.); doina.tofolean@365.univ-ovidius.ro (D.E.T.); 2 Faculty of General Medicine, “Ovidius” University, 900470 Constanta, Romania; bogdan.cimpineanub@365.univ-ovidius.ro (B.C.); ioan.tofolean@365.univ-ovidius.ro (I.T.T.); 3 Gastroenterology Department, “Sf. Apostol Andrei” Emergency County Hospital, 145 Tomis Blvd., 900591 Constanta, Romania; 4 Department of Internal Medicine, “Sf. Apostol Andrei” Emergency County Hospital, 145 Tomis Blvd., 900591 Constanta, Romania; andreea.purcaru@365.univ-ovidius.ro; 5 Nephrology Department, “Sf. Apostol Andrei” Emergency County Hospital, 145 Tomis Blvd., 900591 Constanta, Romania; 6 Pneumology Department, Pneumo-Phthisiology Hospital Palazu Mare, 40 Santinelei Street, 900002 Constanta, Romania; 7 Faculty of Psychology and Educational Sciences, “Ovidius” University, 900470 Constanta, Romania; rodica-gabriela.enache@365.univ-ovidius.ro; 8 Pneumology Department, “Sf. Apostol Andrei” Emergency County Hospital, 145 Tomis Blvd., 900591 Constanta, Romania; 9 Nephrology Department, Clinical Emergency County Hospital Saint John the New in Suceava, 720229 Suceava, Romania; cordos.monica@gmail.com; 10 Department XIII, Discipline of Infectious Diseases, “Victor Babes” University of Medicine and Pharmacy Timisoara, 2 Eftimie Murgu Square, 300041 Timisoara, Romania; alexandra.mocanu@umft.ro; 11 Faculty of Medicine, Titu Maiorescu University, 040051 Bucharest, Romania; alexandrescu_daria@yahoo.com

**Keywords:** chronic obstructive pulmonary disease, multivariable regression, DASS-21, psychological distress, GOLD classification, Bayesian analysis

## Abstract

Background: Psychological distress is a common comorbidity in chronic obstructive pulmonary disease (COPD), yet its relationship with disease severity remains incompletely understood. This study aimed to assess depression, anxiety, and stress using the Depression Anxiety Stress Scales–21 (DASS-21) and to examine their distribution across COPD severity stages. Methods: This multicenter, cross-sectional observational study included 285 clinically stable COPD patients enrolled between 2023 and 2025. COPD severity was classified according to Global Initiative for Chronic Obstructive Lung Disease (GOLD) criteria. Multinomial and binary logistic regression models were constructed to identify independent predictors of COPD severity and clinically significant psychological distress, adjusting for demographic and clinical covariates. Bayesian independent sample analyses and ANOVA effect size estimates were additionally performed. Results: Smoking exposure was independently associated with advanced COPD stages (GOLD 4 vs. GOLD 1–3: aOR 1.05, *p* < 0.001), as was dyspnea severity (mMRC: aOR 14.66, *p* < 0.001). In multivariable models examining psychological outcomes, COPD severity was not independently associated with clinically significant depression (*p* = 0.899), anxiety (*p* = 0.460), or stress (*p* = 0.843). In contrast, symptom burden measured using the COPD Assessment Test (CAT) score was consistently associated with depression (aOR 1.133, *p* < 0.001), anxiety (aOR 1.179, *p* < 0.001), and stress (aOR 1.144, *p* < 0.001). ANOVA effect sizes across GOLD stages were small (η^2^ ≤ 0.047), and Bayesian analyses provided moderate to strong evidence supporting minimal differences in DASS-21-R scores between severity groups. Conclusions: Psychological distress is prevalent across all COPD severity stages and is not independently determined by airflow limitation. Symptom burden rather than spirometric severity appears to be more closely associated with emotional outcomes.

## 1. Introduction

COPD is a progressive respiratory disease characterized by persistent airflow limitation and chronic inflammatory changes in the airways [1,2,3]. Although traditionally defined by spirometric impairment, COPD is now widely recognized as a complex and multidimensional condition extending beyond pulmonary dysfunction. Extrapulmonary manifestations substantially contribute to morbidity, reduced quality of life, and increased healthcare utilization [4,5,6].

**Figure 1:**
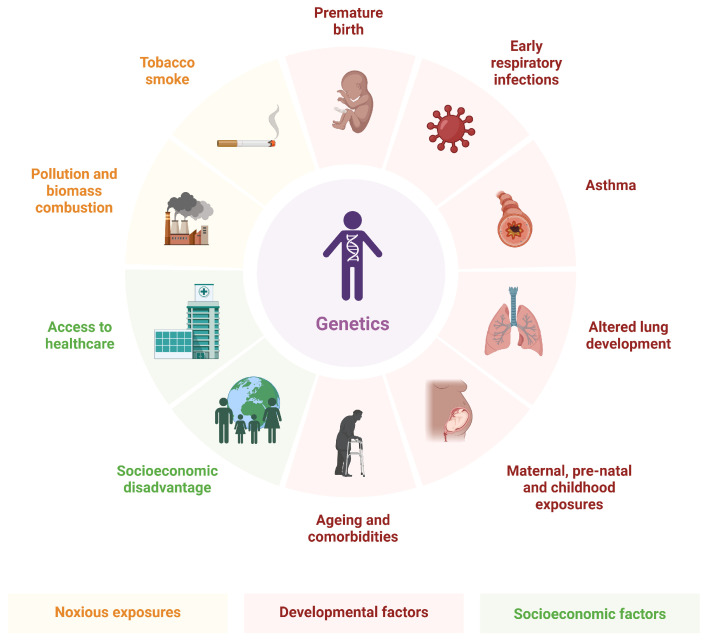
Risk factors for COPD. Created in BioRender. Twakor, A. (2026), https://BioRender.com/jtibn1q (accessed on 25 January 2026) [10].

Among these systemic manifestations, psychological distress, particularly depression, anxiety, and stress, has emerged as a frequent and clinically relevant comorbidity in patients with COPD [7,8,9]. Emotional disturbances are increasingly understood not merely as secondary reactions to physical limitation, but as integral components of disease burden. Figure 1 shows the risk factors for COPD.

Previous studies have shown that psychological symptoms correlate more strongly with perceived symptom burden, dyspnea severity, and functional limitation than with spirometric parameters alone [11,12]. Psychological distress may amplify dyspnea perception, impair coping strategies, reduce treatment adherence, and increase susceptibility to acute exacerbations, thereby establishing a bidirectional interaction between mental health and disease outcomes [13,14,15]. Moreover, large cohort investigations have highlighted the considerable heterogeneity in COPD phenotypes, suggesting that psychosocial factors may influence clinical trajectories independently of structural lung impairment [16,17,18].

**Figure 2:**
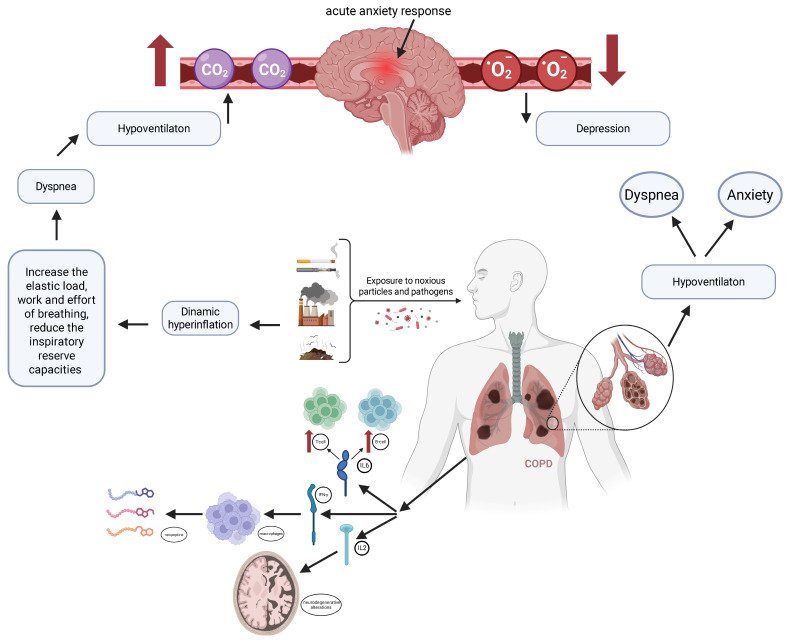
Pathophysiological interplay between respiratory dysfunction, systemic inflammation, and neuropsychological manifestations in COPD. Created in BioRender. Twakor, A. (2026), https://BioRender.com/bohmj9f (accessed on 25 January 2026) [10].

Figure 2 illustrates the bidirectional mechanisms linking COPD with anxiety and depression.

Exposure to noxious particles and pathogens promotes airway injury, leading to airflow limitation and dynamic hyperinflation. The increased elastic load and work of breathing reduce the inspiratory reserve capacity, resulting in dyspnea and hypoventilation. Hypoventilation contributes to carbon dioxide retention (↑CO_2_) and reduced oxygen levels (↓O_2_), which may trigger an acute anxiety response and contribute to depressive symptoms [19].

Simultaneously, persistent pulmonary inflammation induces systemic immune activation, characterized by increased T-cell and B-cell activity and elevated pro-inflammatory cytokines, including IL-6, IFN-γ, and IL-2 [20]. Activated macrophages and inflammatory mediators may influence neurochemical pathways and increase blood–brain barrier permeability, facilitating central nervous system involvement. These processes are associated with neurodegenerative changes, motor and cognitive dysfunction, and mood disturbances [21]. A reciprocal cycle is highlighted between dyspnea, anxiety, and hypoventilation, reinforcing symptom amplification.

**Figure 3:**
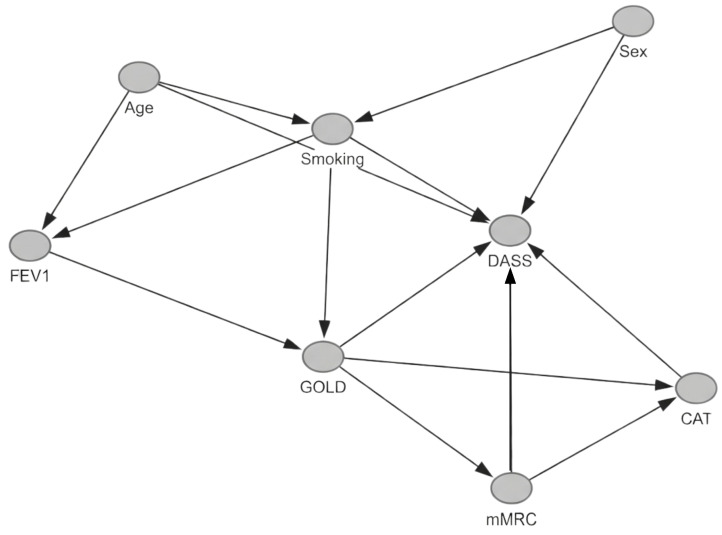
Directed acyclic graph illustrating the hypothesized causal relationships between COPD severity and psychological distress. Created in [22].

Figure 3 shows the demographic factors (age and sex) that are modeled as common causes of smoking exposure and psychological distress.

Smoking exposure influences lung function (FEV1) and the GOLD stage, while the GOLD stage affects dyspnea severity, overall symptom burden (CAT), and psychological distress. Symptom burden variables (mMRC and CAT) are conceptualized as potential mediators in the pathway between disease severity and emotional outcomes [23].

According to GOLD criteria, COPD is diagnosed based on persistent airflow limitation confirmed by post-bronchodilator spirometry, demonstrating a forced expiratory volume in one second-to-forced vital capacity ratio (FEV_1_/FVC) < 0.70 [24].

Therefore, the present study aimed to assess depression, anxiety, and stress using DASS-21 in a well-characterized COPD cohort and to explore their distribution across GOLD severity stages.

## 2. Materials and Methods

### 2.1. Study Design and Population

This multicenter, observational study was conducted in two clinical settings in Constanța, Romania: the Pneumophthisiology Hospital “Palazu Mare” and the County Emergency Hospital “Sf. Apostol Andrei”, including Medical Clinic 1 and Medical Clinic 2. A total of 285 adult patients diagnosed with chronic obstructive pulmonary disease (COPD) were consecutively evaluated between 2023 and 2025. The study was performed in accordance with the Declaration of Helsinki and received approval from the Ethics Committees of the Pneumophthisiology Hospital “Palazu Mare” (approval code 2400/21.03.2023) and the County Emergency Hospital “Sf. Apostol Andrei” (approval code 24907/26.04.2023).

### 2.2. Inclusion and Exclusion Criteria

Patients were eligible if they were ≥18 years of age, hospitalized and had a confirmed diagnosis of COPD based on GOLD criteria, defined by post-bronchodilator spirometry demonstrating a FEV_1_/FVC ratio < 0.70. Only Romanian-speaking patients able to complete questionnaires were included. Complete psychological assessment data using the Depression Anxiety Stress Scales–21 were required for study participation.

Exclusion criteria comprised the presence of other primary respiratory diseases, including asthma, interstitial lung disease, bronchiectasis, pulmonary fibrosis, or active pulmonary tuberculosis. Patients with acute COPD exacerbations, severe cognitive impairment, or psychiatric disorders interfering with reliable questionnaire completion, as well as those with incomplete DASS-21 data or a refusal to provide informed consent, were excluded.

### 2.3. Psychological and Clinical Assessment

Psychological distress was assessed using the Depression Anxiety Stress Scales—21 items (DASS-21), originally developed by Lovibond and Lovibond in 1995 [24]. The Romanian validated version used in this study was the DASS 21-R, adapted and standardized for the Romanian population by Perțe (coordinator) and Albu, published by ASCR (Cluj-Napoca, 2011) [25]. The instrument evaluates three dimensions, depression, anxiety, and stress, each consisting of seven items. Participants rated the frequency/severity of symptoms experienced over the previous week using a 4-point Likert scale. The questionnaire was administered in paper format during clinical evaluation under medical supervision.

Internal consistency in the Romanian standardization sample (*N* = 1053) demonstrated a high reliability, with Cronbach’s alpha coefficients of 0.851 for depression, 0.855 for anxiety, and 0.865 for stress [26]. In the present study, internal consistency was recalculated, confirming an adequate reliability for all three subscales (Cronbach’s α > 0.80).

A directed acyclic graph was constructed to explicitly represent the hypothesized causal relationships between demographic factors, smoking exposure, lung function, COPD severity, symptom burden, and psychological distress [22].

The diagnosis of COPD required post-bronchodilator spirometry, demonstrating a forced expiratory volume in one second-to-forced vital capacity ratio (FEV_1_/FVC) < 0.70 [24]. Disease severity (GOLD stages 1–4) was determined based on FEV_1_ (% predicted), with stage 1 defined as FEV_1_ ≥ 80%, stage 2 as 50–79%, stage 3 as 30–49%, and stage 4 as <30% of predicted values. For complementary analyses, the GOLD stage was dichotomized as GOLD 4 versus GOLD 1–3. This categorization was chosen because GOLD stage 4 represents a very severe airflow limitation and reflects the most advanced stage of COPD. GOLD classification was determined by certified pulmonologists based on post-bronchodilator spirometry results obtained during hospitalization, following standardized protocols.

Dyspnea severity was assessed using the modified Medical Research Council (mMRC) dyspnea scale, which ranges from 0 to 4, with higher scores indicating greater dyspnea-related functional limitation [27]. Symptom burden was evaluated using CAT, an eight-item questionnaire designed to measure the impact of COPD on health status. CAT scores range from 0 to 40, with higher scores reflecting a greater symptom burden and disease impact [28].

### 2.4. Statistical Analysis

Statistical analyses were performed using SPSS version 29 (IBM Corp., Armonk, NY, USA) version 29, with significance set at *p* < 0.05 [29]. In accordance with STROBE recommendations [30], regression-based modeling was implemented to account for potential confounding. GOLD stage (1–4) was analyzed as the primary outcome using multinomial logistic regression. For selected analyses, the study population was dichotomized as GOLD stage 4 versus GOLD stages 1–3. This cutoff was adopted because GOLD stage 4 represents a very severe airflow limitation and reflects the most advanced stage of COPD, typically associated with marked functional impairment and an increased risk of complications.

Psychological distress domains were categorized according to clinically relevant thresholds and analyzed as dependent variables in separate multivariable logistic regression models. Covariates were selected a priori based on the established literature regarding determinants of COPD severity and psychological outcomes and included age, sex, cumulative smoking exposure (pack-years), current smoking status, dyspnea severity assessed using mMRC, symptom burden measured using CAT score, FEV1 (% predicted), and long-term oxygen therapy. Distributional assumptions for continuous psychological variables were assessed through a visual inspection of histograms and Q–Q plots prior to inferential testing.

Adjusted odds ratios (aORs) with 95% confidence intervals were calculated [31]. Model performance was evaluated using omnibus tests of model coefficients, Nagelkerke R^2^ estimates, and Hosmer–Lemeshow goodness-of-fit tests [32]. Multicollinearity diagnostics were assessed using variance inflation factors, and model assumptions were verified prior to interpretation [33].

In addition to frequentist analyses, Bayesian independent sample analyses [34] were conducted to compare GOLD severity groups across demographic, clinical, and psychological variables. Bayes factors (BF_01_) were calculated using the Rouder method [35]. Posterior distributions of mean differences were estimated using weakly informative priors centered at zero to complement the probabilistic interpretation of effect sizes [36].

**Figure 4:**
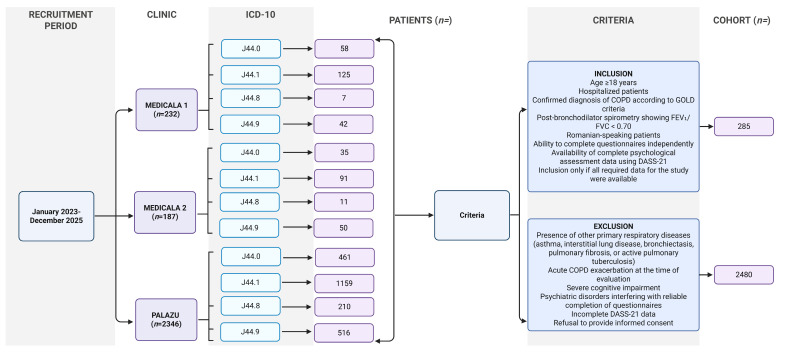
Flow diagram of patient recruitment and selection process. Created in BioRender. Twakor, A. (2026), https://BioRender.com/myvdk9v (accessed on 25 January 2026) [10]. The following ICD-10 codes were used: J44.0—COPD with acute lower respiratory infection; J44.1—COPD with acute exacerbation, unspecified; J44.8—other specified COPD; and J44.9—COPD, unspecified [37].

As this was an observational study with consecutive enrollment, no a priori power calculation was performed. A post hoc evaluation based on regression modeling principles was conducted. The total sample included 285 participants, with 81 individuals in the GOLD 4 group (Figure 4).

Seven predictors were included in the multivariable models, yielding an events-per-variable (EPV) ratio of 81/7 ≈ 11.6, exceeding the recommended threshold of 10 for stable alogistic regression estimates. Under standard assumptions (α = 0.05, power = 0.80), the sample size allows the detection of moderate effect sizes (OR ≈ 1.7–1.9), but smaller associations may not be detectable.

## 3. Results

### 3.1. Population Characteristics

Table 1 and Table 2 summarize the demographic, clinical, and psychological characteristics of the study population stratified according to COPD severity. In addition to standard demographic variables such as sex and obesity status, the tables include clinically relevant indicators including smoking history (pack-years, current smoking, and smoking cessation), dyspnea severity assessed using the mMRC scale, symptom burden measured using CAT, and the need for long-term oxygen therapy.

The study cohort comprised 285 patients recruited from three clinical centers, with the largest proportions originating from Medicala 2 (42.8%) and Palazu (39.6%), followed by Medicala 1 (17.5%). The population was predominantly male (76.1%), and obesity was present in 38.6% of participants. A substantial proportion of patients had respiratory failure (61.4%), while other pulmonary diseases were reported in 34.4%, pulmonary embolism in 17.2%, heart disease in 26.0%, and cardiac failure in 39.6%. Diabetes was highly prevalent, affecting nearly half of the cohort (49.8%). Atrial fibrillation or flutter was identified in 15.1% of cases, and elevated blood pressure was common (72.3%). The vast majority of participants were current smokers (93.7%), although only 16.1% reported smoking cessation. Long-term oxygen therapy was used by 45.6% of patients. Regarding treatment adherence, 71.6% were classified as adherent according to the MARS scale, whereas 28.4% were non-adherent.

The mean age of the cohort was 66 years (95% CI: 65–67), with values ranging from 34 to 96 years (Table 2).

The average GOLD stage was 3 (range 1–4). The mean FEV_1_ was 45.6% (95% CI: 43.1–48.1), and the mean FVC was 72.5% (95% CI: 69.7–75.3). The mean pack-year index was 37 (range 0–100). The mean peripheral oxygen saturation (SpO_2_) was 93% (95% CI: 92–93%), and the mean ventricular rate was 92 beats per minute (95% CI: 90–94). The mean mMRC score was 3 (range 0–4), and the mean CAT score was 23 (range 7–37). The WHO-5 well-being index had a mean value of 51% (95% CI: 49–53%). The median scores for DASS-21 depression, anxiety, and stress were 2 across domains, with observed ranges between 0 and 4. Table S1 from the Supplementary Materials contains population characteristics according to the GOLD severity stage.

### 3.2. Logistic Regression Analysis

To identify independent factors associated with COPD severity, a multinomial logistic regression model was constructed using GOLD stage 1 as the reference category (Table 3). The model included age, sex, smoking exposure (pack-years), dyspnea severity, symptom burden, FEV1 (% predicted), smoking status, and long-term oxygen therapy as covariates. Adjusted odds ratios (aORs) with 95% confidence intervals were calculated for GOLD stages 2, 3, and 4 relative to stage 1.

After the adjustment for relevant clinical and demographic variables, smoking exposure (pack-years) was independently associated with a higher COPD severity across all comparisons. Each additional pack-year increased the odds of belonging to GOLD stage 2 (aOR 1.15, 95% CI 1.02–1.30), stage 3 (aOR 1.17, 95% CI 1.03–1.32), and stage 4 (aOR 1.22, 95% CI 1.08–1.39) relative to stage 1. Dyspnea severity (mMRC) demonstrated a strong and consistent association with advanced disease stages (*p* < 0.001 across comparisons), reflecting the close relationship between functional limitation and disease progression. Higher CAT scores were also significantly associated with GOLD stages 3 and 4, although the direction of the association suggests an overlap between symptom burden and severity classification. In contrast, age, sex, FEV1, smoking status, and long-term oxygen therapy were not independently associated with the GOLD stage after multivariable adjustment.

We also ran a multivariable binary logistic regression model using GOLD stage 4 as the outcome variable (reference category: GOLD stages 1–3). The model included age, sex, pack-year index, current smoking status, mMRC, CAT score, FEV1, and long-term oxygen therapy as covariates (Table 4). Adjusted odds ratios with 95% confidence intervals were estimated to quantify the association between each predictor and the likelihood of belonging to the GOLD stage 4 group.

Binary logistic regression analysis identified cumulative smoking exposure and dyspnea severity as independent predictors of GOLD 4 disease. Each additional pack-year increased the odds of very severe COPD by 4.7% (aOR 1.05, 95% CI 1.02–1.07, *p* < 0.001). Dyspnea severity demonstrated a strong independent association (aOR 14.66, 95% CI 6.43–33.43, *p* < 0.001). Age showed a modest inverse association (aOR 0.96, *p* = 0.048). Sex, smoking status, CAT score, FEV1, and oxygen therapy were not independently associated with GOLD 4 after multivariable adjustment.

**Figure 5:**
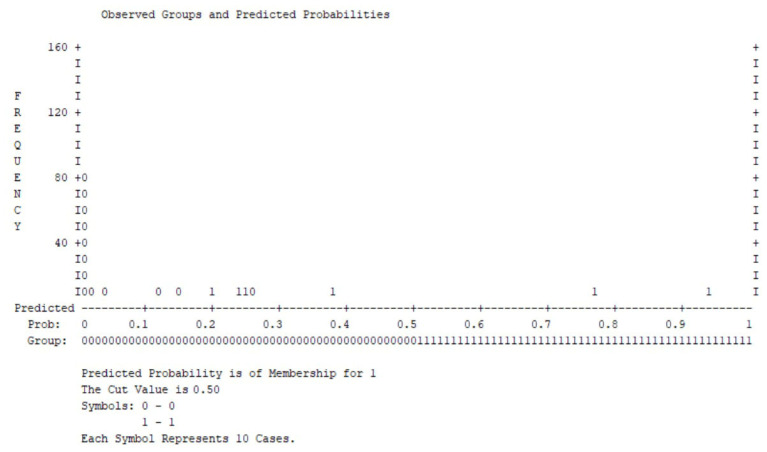
Observed groups and predicted probabilities for clinically significant psychological distress (cut-off = 0.50).

Figure 5 displays the distribution of predicted probabilities for clinically significant psychological distress derived from the multivariable logistic regression model. A classification threshold of 0.50 was applied.

Most patients without elevated distress cluster at lower predicted probabilities, whereas individuals with clinically significant symptoms are predominantly distributed toward higher probability values, indicating an acceptable separation between groups. This visual discrimination is consistent with the overall model performance, which demonstrated statistical significance in the omnibus test, adequate explained variance (Nagelkerke R^2^), and good calibration according to the Hosmer–Lemeshow test (*p* > 0.05).

We further constructed a multivariable binary logistic regression model using dichotomized DASS depression scores (≥2 vs. 0–1) as the dependent variable (Table 5). The model included COPD severity (GOLD stage 4 vs. GOLD stages 1–3), age, sex, cumulative smoking exposure, current smoking status, dyspnea severity, symptom burden, FEV1, and long-term oxygen therapy as covariates.

In the adjusted model, the CAT score emerged as the only independent predictor of clinically significant psychological distress. Each one-point increase in the CAT score was associated with a 13.3% increase in the odds of elevated DASS symptoms (aOR 1.133, 95% CI 1.081–1.188, *p* < 0.001). Age showed a modest inverse association, with increasing age slightly reducing the likelihood of psychological distress (aOR 0.971, 95% CI 0.943–0.999, *p* = 0.042). In contrast, COPD severity (GOLD stage 4), sex, cumulative smoking exposure, smoking status, dyspnea severity (mMRC), FEV1, and long-term oxygen therapy were not independently associated with psychological distress after multivariable adjustment.

**Figure 6:**
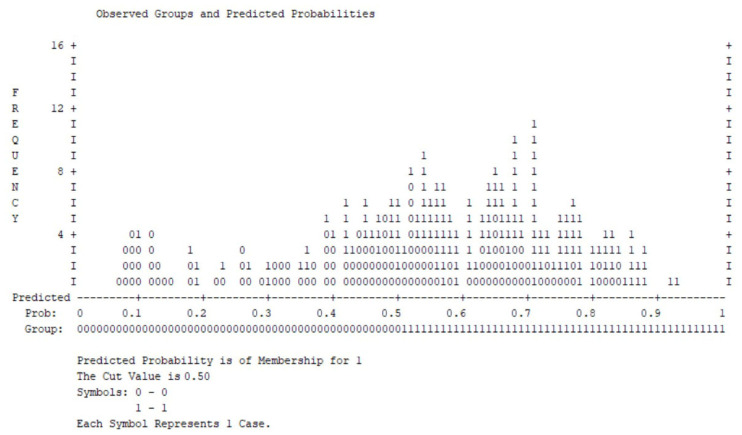
Observed groups and predicted probabilities for clinically significant depressive symptoms (DASS depression ≥ 2; cut-off = 0.50).

Most patients without clinically significant depression cluster at lower predicted probabilities, while cases with elevated depressive symptoms tend to accumulate at higher probability values (Figure 6).

Although some overlap is present around the classification threshold, the distribution indicates an acceptable discrimination between outcome groups. This visual pattern is consistent with the overall statistical performance of the model, including significant omnibus testing, adequate explained variance (Nagelkerke R^2^), and satisfactory calibration according to the Hosmer–Lemeshow goodness-of-fit test (*p* > 0.05), supporting the model’s validity in identifying depressive symptom burden within this COPD cohort.

Based on the same model, next we identified independent predictors of clinically significant anxiety symptoms (Figure 6 and Table 6).

The CAT score was the only independent predictor of clinically significant anxiety symptoms. Each one-point increase in the CAT score was associated with a 17.9% increase in the odds of elevated anxiety levels (aOR 1.179, 95% CI 1.120–1.241, *p* < 0.001). COPD severity (GOLD stage 4), age, sex, cumulative smoking exposure, smoking status, dyspnea severity (mMRC), FEV1, and long-term oxygen therapy were not independently associated with anxiety symptoms after multivariable adjustment.

**Figure 7:**
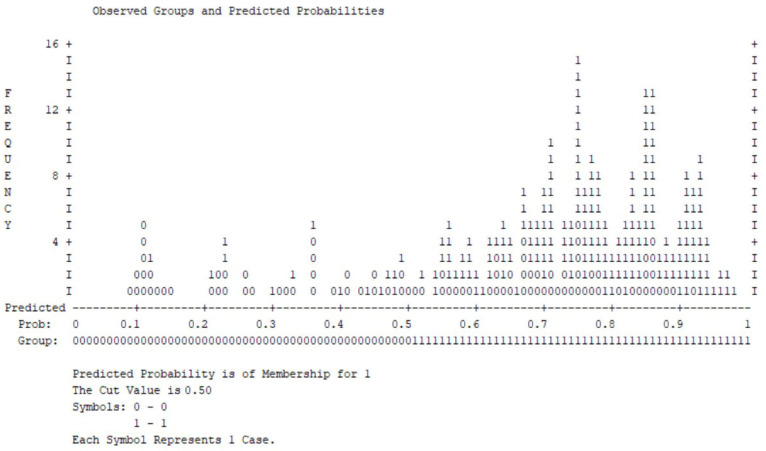
Observed groups and predicted probabilities for clinically significant anxiety symptoms (DASS anxiety ≥ 2; cut-off = 0.50).

Figure 7 shows the distribution of predicted probabilities for clinically significant anxiety symptoms. Most individuals without clinically significant anxiety cluster at lower predicted probabilities, while cases with elevated anxiety symptoms are predominantly distributed toward higher probability values.

Although a partial overlap is observed around the decision threshold, the overall distribution indicates an acceptable discrimination between outcome groups.

The model included in Table 7 shows COPD severity (GOLD stage 4 vs. GOLD stages 1–3), age, sex, cumulative smoking exposure, current smoking status, dyspnea severity, symptom burden, FEV1, and long-term oxygen therapy as covariates.

In the adjusted model, the symptom burden measured using the CAT score emerged as the only strong independent predictor of clinically significant stress symptoms. Each one-point increase in the CAT score was associated with a 14.4% increase in the odds of elevated stress levels (aOR 1.144, 95% CI 1.090–1.199, *p* < 0.001). Age demonstrated a modest inverse association with stress symptoms, with increasing age associated with slightly lower odds of clinically significant stress (aOR 0.967, 95% CI 0.938–0.997, *p* = 0.032). COPD severity (GOLD stage 4), sex, cumulative smoking exposure, smoking status, dyspnea severity (mMRC), FEV1, and long-term oxygen therapy were not independently associated with stress symptoms after multivariable adjustment.

**Figure 8:**
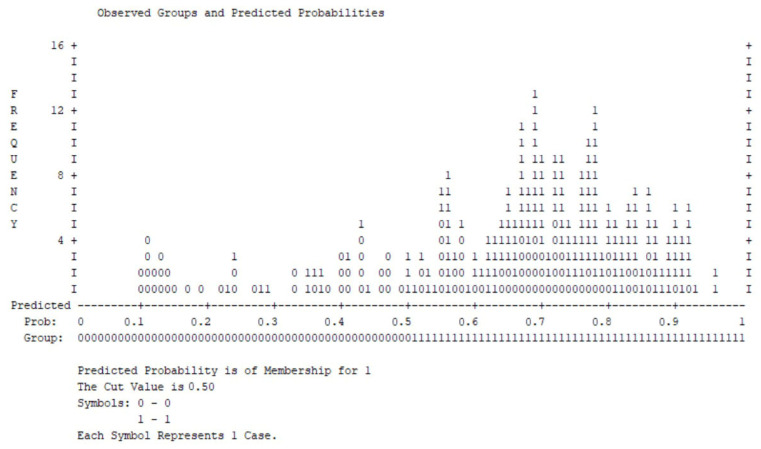
Observed groups and predicted probabilities for clinically significant stress symptoms (DASS stress ≥ 2; cut-off = 0.50).

Figure 8 displays the distribution of predicted probabilities for clinically significant stress symptoms.

Most patients without clinically significant stress cluster at lower predicted probabilities, whereas cases with elevated stress symptoms are primarily distributed toward higher probability values. Although some overlap is evident around the decision threshold, the overall distribution demonstrates an acceptable discriminative ability.

Across the three multivariable logistic regression models examining clinically significant depression, anxiety, and stress symptoms, a consistent pattern emerged. COPD severity (GOLD stage 4 vs. GOLD stages 1–3) was not independently associated with any psychological distress domain after the adjustment for demographic and clinical covariates. Similarly, cumulative smoking exposure, current smoking status, dyspnea severity (mMRC), FEV1 (% predicted), and long-term oxygen therapy did not demonstrate independent associations with depressive, anxiety, or stress symptoms. In contrast, the symptom burden measured using the CAT score was a strong and consistent predictor across all models. Each one-point increase in the CAT score was associated with increased odds of depression (aOR 1.133, *p* < 0.001), anxiety (aOR 1.179, *p* < 0.001), and stress (aOR 1.144, *p* < 0.001). Age showed a modest inverse association with depression and stress but was not significant for anxiety.

### 3.3. Bayesian Analysis

Bayesian independent sample analyses were performed to compare patients with GOLD < 4 and GOLD = 4 across demographic, clinical, and psychological variables. Bayesian inference was conducted using the Rouder method for independent groups, assuming unequal variances (Table 8). Graphical representations of the Bayesian posterior distributions for individual variables are provided in Supplementary Figures S1–S9.

Bayesian independent sample analysis revealed decisive evidence for differences between GOLD < 4 and GOLD = 4 groups in cumulative smoking exposure and mMRC, with Bayes factors strongly favoring the existence of a difference between the two GOLD groups in the variable being tested. Oxygen therapy demonstrated only weak evidence supporting a difference between groups. In contrast, demographic variables, CAT scores, and all DASS-21 domains (depression, anxiety, and stress) yielded Bayes factors indicating moderate evidence that there is no difference between the GOLD groups. Table 9 includes descriptive statistics for psychological variables.

Patients with GOLD = 4 showed a significantly higher pack-year index and more severe dyspnea, as reflected by elevated mMRC scores, with Bayes factors indicating decisive evidence for group differences. Oxygen therapy also differed between groups, although the Bayes factor suggested only weak evidence supporting this difference. For demographic variables, smoking status, CAT scores, and psychological measures assessed using DASS-21, Bayes factors provided moderate to strong evidence for the absence of meaningful group differences. Notably, depression, anxiety, and stress scores did not differ significantly between GOLD stages, reinforcing the observation that psychological distress in COPD is not solely dependent on disease severity.

**Figure 9:**
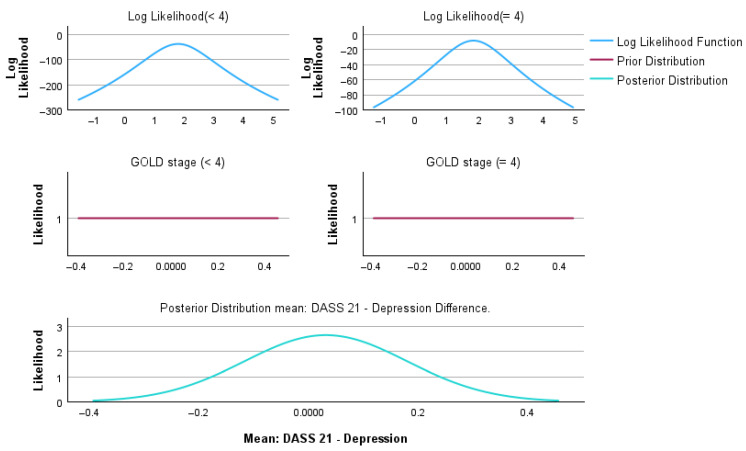
Bayesian estimation of the mean difference in DASS-21 depression scores between COPD severity groups (GOLD < 4 vs. GOLD = 4).

Figure 9 illustrates the Bayesian framework used to estimate the mean difference in DASS-21 depression scores between patients with GOLD < 4 and GOLD = 4 COPD. Further variables analyzed are included in Supplementary Materials.

The upper panels display the log-likelihood functions for each severity group, reflecting the observed data and indicating substantial overlap between distributions. The middle panels show the prior distributions, specified as weakly informative and centered around zero, reflecting the absence of strong a priori assumptions regarding group differences. The lower panel presents the resulting posterior distribution for the mean difference in DASS-21 depression scores. The posterior distribution is centered close to zero and exhibits a relatively narrow spread, indicating that the most credible values of the group difference cluster around minimal effect sizes. This pattern suggests a high probability that differences in depressive symptom severity between COPD severity groups are small or clinically negligible. The Bayesian posterior inference is consistent with the Bayes factor results, supporting the conclusion that depressive symptoms, as measured using DASS-21, are prevalent across COPD stages rather than being strongly driven by disease severity.

**Figure 10:**
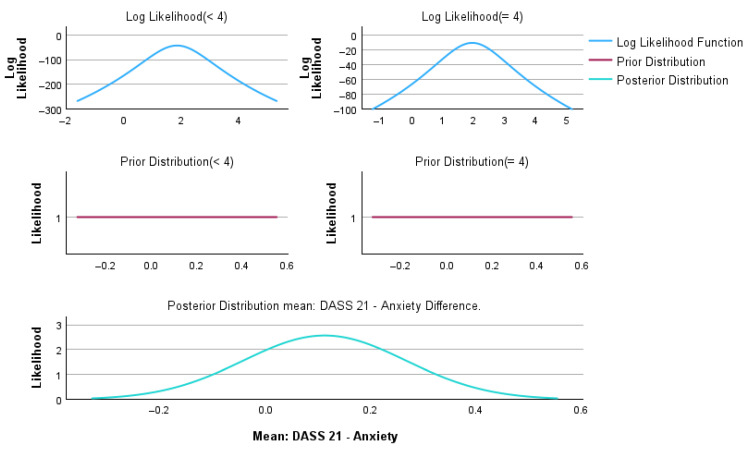
Bayesian estimation of the mean difference in DASS-21 anxiety scores between COPD severity groups (GOLD < 4 vs. GOLD = 4).

Figure 10 presents the Bayesian estimation of the mean difference in DASS-21 anxiety scores between patients with GOLD < 4 and GOLD = 4 COPD.

The log-likelihood functions show a marked overlap between groups, indicating comparable anxiety score distributions. Using weakly informative priors centered at zero, the posterior distribution of the mean difference was centered at a small positive value (mean difference = 0.11), consistent with the frequentist results (t = 0.705, *p* = 0.482). The posterior density was narrowly distributed around zero, and the Bayes factor (BF_01_ = 7.625) provided moderate evidence supporting the absence of a clinically meaningful difference between groups. These Bayesian findings corroborate the results reported in Table 2 and indicate that anxiety symptoms, as measured by DASS-21, are similarly prevalent in patients with moderate and very severe COPD.

**Figure 11:**
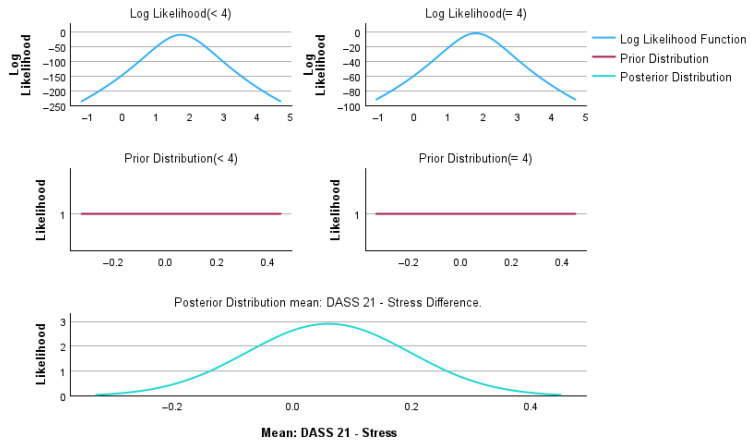
Bayesian estimation of the mean difference in DASS-21 stress scores between COPD severity groups (GOLD < 4 vs. GOLD = 4).

Figure 11 illustrates the Bayesian estimation of the mean difference in DASS-21 stress scores between patients with GOLD < 4 and GOLD = 4 COPD.

The log-likelihood functions demonstrate a substantial overlap between groups, indicating similar stress score distributions. Weakly informative priors centered at zero were applied, and the resulting posterior distribution of the mean difference was centered close to zero (mean difference = 0.06). This finding is consistent with the frequentist analysis (t = 0.433, *p* = 0.665) and the Bayes factor results (BF_01_ = 8.857). The posterior density shows that the most credible values correspond to very small effect sizes, suggesting the absence of a clinically meaningful difference in stress levels between COPD severity groups.

Table 10 presents descriptive statistics for DASS-21 depression, anxiety, and stress scores stratified by GOLD stages, along with overall estimates for the entire cohort.

For depression, mean scores ranged from 1.48 in GOLD stage 2 to 2.10 in GOLD stage 1, with an overall mean of 1.79 (95% CI: 1.66–1.93). Anxiety scores showed a similar pattern, with mean values varying between 1.55 (GOLD 2) and 2.24 (GOLD 1), and a total cohort mean of 1.87 (95% CI: 1.73–2.01). Stress scores ranged from 1.36 in GOLD stage 2 to 1.94 in GOLD stage 3, yielding an overall mean of 1.75 (95% CI: 1.63–1.87).

Across all three psychological domains, confidence intervals overlapped substantially between GOLD stages, indicating comparable levels of depressive, anxiety, and stress symptoms across disease severity categories. Fixed-effect model estimates closely matched the total sample means, while random-effect models showed slightly wider confidence intervals, reflecting between-group variability.

Table 11 reports effect size estimates derived from one-way ANOVA analyses [22] examining differences in DASS-21 depression, anxiety, and stress scores across GOLD stages. Assumptions of homogeneity of variances were assessed using Levene’s test, with detailed results provided in Supplementary Table S2.

For depression, the effect size was small, with an eta-squared value of 0.028 (95% CI: 0.000–0.067), and similarly low estimates for epsilon-squared and omega-squared (fixed-effect ω^2^ = 0.017). Anxiety demonstrated an almost identical pattern, with an eta-squared of 0.028 (95% CI: 0.000–0.068) and omega-squared values indicating minimal explained variance. The full one-way ANOVA results across GOLD stages are presented in Supplementary Table S3.

Stress scores showed slightly higher effect size estimates, with an eta-squared of 0.047 (95% CI: 0.006–0.095) and a fixed-effect omega-squared of 0.037, suggesting a small but measurable effect of GOLD stage on stress levels. However, random-effect omega-squared estimates remained low across all psychological domains (ω^2^ ≤ 0.013), indicating limited between-group variability attributable to disease severity.

**Figure 12:**
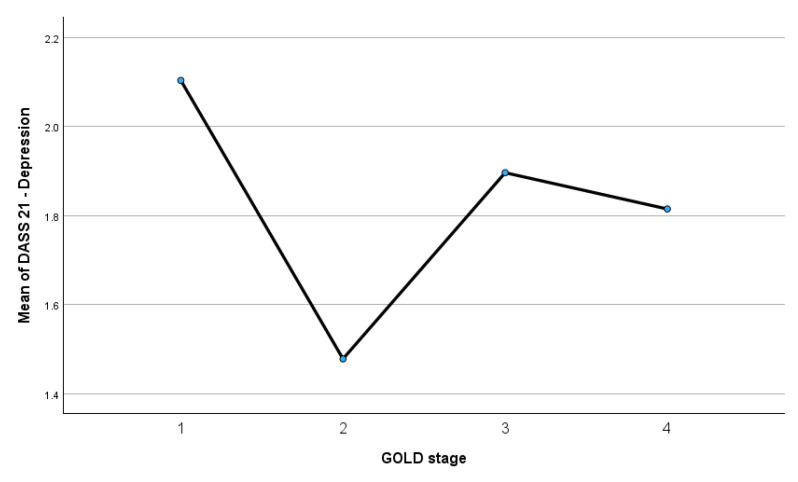
Mean DASS-21 depression scores across GOLD stages.

Figure 12 illustrates the distribution of mean DASS-21 depression scores across GOLD stages 1 to 4.

The mean depression scores were highest in GOLD stage 1 (mean = 2.10) and lowest in GOLD stage 2 (mean = 1.48), followed by a moderate increase in GOLD stage 3 (mean = 1.90) and a slight decrease in GOLD stage 4 (mean = 1.81). Although numerical variations are observed across stages, the overall pattern does not demonstrate a consistent linear increase in depressive symptoms with advancing COPD severity. This visual trend aligns with the ANOVA findings, which indicated a small effect size for depression (η^2^ = 0.028), and with Bayesian analyses showing evidence in favor of the absence of meaningful differences between severity groups.

**Figure 13:**
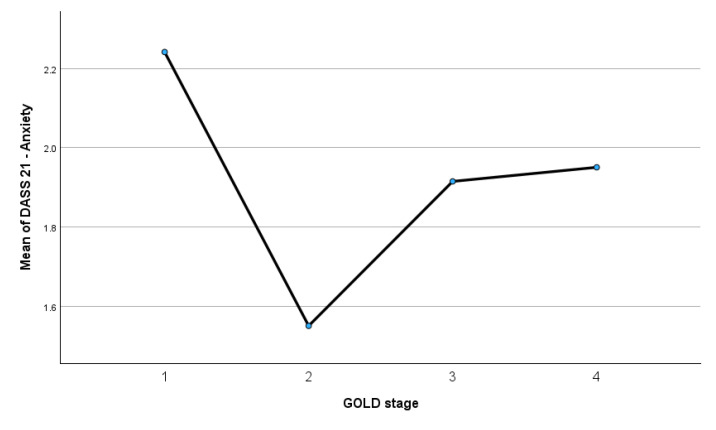
Mean DASS-21 anxiety scores across GOLD stages.

Figure 13 depicts the mean DASS-21 anxiety scores across GOLD stages 1 to 4.

Anxiety levels were highest in GOLD stage 1 (mean = 2.24) and lowest in GOLD stage 2 (mean = 1.55), followed by an increase in GOLD stage 3 (mean = 1.92) and a slight further increase in GOLD stage 4 (mean = 1.95). Despite these numerical fluctuations, the pattern does not indicate a progressive increase in anxiety symptoms with advancing COPD severity. This visual trend is consistent with the one-way ANOVA results, which demonstrated a small effect size for anxiety (η^2^ = 0.028), and with Bayesian analyses providing moderate evidence for this (BF_01_ = 7.625).

**Figure 14:**
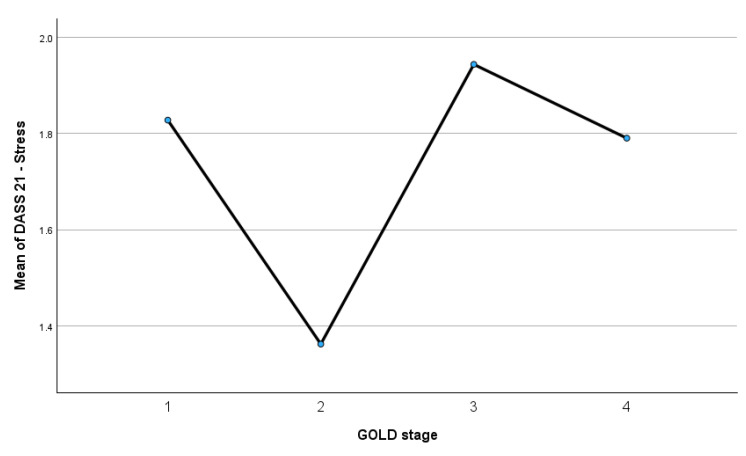
Mean DASS-21 stress scores across GOLD stages.

Figure 14 illustrates the mean DASS-21 stress scores across GOLD stages 1 to 4.

Stress levels were highest in GOLD stage 3 (mean = 1.94) and lowest in GOLD stage 2 (mean = 1.36), with intermediate values observed in GOLD stage 1 (mean = 1.83) and GOLD stage 4 (mean = 1.79). Although a variability in stress scores is evident across GOLD stages, the pattern does not demonstrate a monotonic increase in stress with advancing COPD severity.

Overall, depression scores ranged from 1.48 in GOLD stage 2 to 2.10 in GOLD stage 1, anxiety scores ranged from 1.55 to 2.24, and stress scores ranged from 1.36 to 1.94. Across all three psychological domains, numerical fluctuations were observed between GOLD stages; however, no consistent linear trend with increasing disease severity was evident. These visual patterns are consistent with the statistical analyses, which demonstrated small effect sizes for depression (η^2^ = 0.028), anxiety (η^2^ = 0.028), and stress (η^2^ = 0.047), as well as Bayesian evidence favoring the absence of meaningful differences between severity groups.

## 4. Discussion

In this cohort, psychological distress measured using DASS-21 did not differ independently across GOLD severity stages after adjustment for relevant covariates. Although symptom burden and smoking exposure were associated with more severe disease, the adjusted regression analyses indicate that psychological distress is not directly determined by spirometric severity. Similar to our findings, several prior investigations suggested that factors other than airflow limitation per se may play a stronger role in emotional outcomes [38,39,40,41].

Consistent with our results, Crisan et al. reported that, while severe COPD was associated with differences in some psychological measures, the relationship between lung function impairment and psychological factors was inconsistent, underscoring the complexity of this association in cross-sectional analyses [42]. Similarly, studies synthesizing the relationship between physical activity and psychological symptoms in COPD have highlighted a heterogeneity in how anxiety and depression relate to disease features such as dyspnea and functional impairment rather than purely spirometric indices [43,44].

Previous work has emphasized the high prevalence of anxiety and depressive symptoms among individuals with COPD compared with the general population or non-COPD cohorts. Narrative reviews report elevated rates of both depression and anxiety, with a substantial clinical impact on morbidity, quality of life, and healthcare utilization [45]. Similarly, broader reviews of psychological distress in COPD have documented large ranges of prevalence and pointed to dyspnea and symptom burden as central to psychological morbidity [46]. Our observations also show that symptom burden, as captured by CAT scores, was more strongly associated with DASS-21 domains than spirometric severity.

The literature also highlights the complex interplay between psychological symptoms and clinical outcomes in COPD, including adverse effects on prognosis, adherence, and functional status. Martínez-Gestoso et al. found high prevalence rates of anxiety and depression in patients hospitalized for acute COPD exacerbations, with depression independently associated with readmission risk and clinical outcomes [47]. Although our study focused on stable outpatients and did not examine exacerbation outcomes, this evidence shows the clinical significance of psychological distress regardless of lung function severity.

The inconsistent relationship between airflow limitation and psychological distress in COPD has also been highlighted in studies examining specific behavioral correlations. For instance, the association between physical activity and anxiety or depression is variable across studies [48].

The current evidence indicates that, while emotional distress is common in COPD and is associated with clinical outcomes, it does not reliably escalate with worsening airflow limitation alone. Rather, distress appears to be influenced by a combination of symptom perception, functional burden, and psychosocial context.

## 5. Limitations

This study has several limitations. First, the design does not allow causal inference between COPD severity and psychological distress. Second, psychological symptoms were assessed using the self-reported DASS-21 questionnaire rather than structured psychiatric interviews, which may introduce reporting bias. Third, although the sample size was adequate for detecting moderate associations, smaller effect sizes may not have been identified. Fourth, participants were recruited from hospital-based settings, which may limit generalizability to community-dwelling COPD populations. Finally, despite multivariable adjustment, residual confounding cannot be entirely excluded.

## 6. Conclusions

In this multicenter observational study, psychological distress assessed using the DASS-21 was prevalent across all COPD severity stages. Although patients with more advanced disease exhibited a greater smoking exposure and functional impairment, multivariable regression and Bayesian analyses consistently demonstrated that COPD severity was not independently associated with clinically significant depression, anxiety, or stress after adjustment for relevant demographic and clinical factors. Instead, the overall symptom burden emerged as the most consistent correlate of psychological distress.

Thus, emotional symptoms in COPD are not solely determined by the degree of airflow limitation but reflect a multidimensional interaction between clinical status, perceived disease impact, and psychosocial factors. Routine psychological screening should therefore be considered across all stages of COPD, rather than being restricted to patients with advanced disease.

## Figures and Tables

**Table 1:** Population characteristics statistics (frequencies).

		N	%
Clinic	Palazu	113	39.6%
	Medicala 1	50	17.5%
	Medicala 2	122	42.8%
Sex	Male	217	76.1%
	Female	68	23.9%
Obesity	No	175	61.4%
	Yes	110	38.6%
Respiratory failure	No	110	38.6%
	Yes	175	61.4%
Other PD	No	187	65.6%
	Yes	98	34.4%
Pulmonary embolism	No	236	82.8%
	Yes	49	17.2%
Heart disease	No	211	74.0%
	Yes	74	26.0%
Cardiac failure	No	172	60.4%
	Yes	113	39.6%
Diabetes	No	143	50.2%
	Yes	142	49.8%
Atrial fibrillation/flutter	No	242	84.9%
	Yes	43	15.1%
BP	No	79	27.7%
	Yes	206	72.3%
Smoker	No	18	6.3%
	Yes	267	93.7%
Smoking cessation	No	239	83.9%
	Yes	46	16.1%
Oxygen therapy	No	155	54.4%
	Yes	130	45.6%
MARS	Non-adherent	81	28.4%
	Adherent	204	71.6%

**Table 2:** Population characteristics statistics.

	Mean	95.0% Upper CL of Mean	95.0% Lower CL of Mean	Standard Error of Mean	Median	Maximum	Minimum
Age	66	67	65	1	67	96	34
GOLD	3	3	3	0	3	4	1
FEV1	45.6	48.1	43.1	1.3	40.0	105.7	15.0
FVC	72.5	75.3	69.7	1.4	69.8	142.2	26.2
Pack-year index	37	40	35	1	34	100	0
SpO2	93%	93%	92%	0%	93%	99%	52%
Ventricular rate	92	94	90	1	94	153	53
mMRC	3	3	2	0	3	4	0
CAT	23	24	23	0	24	37	7
WHO_5	51%	53%	49%	1%	48%	92%	20%
DASS-21 depression	2	2	2	0	2	4	0
DASS-21 anxiety	2	2	2	0	2	4	0
DASS-21 stress	2	2	2	0	2	4	0

**Table 3:** Multinomial logistic regression analysis of factors associated with GOLD severity stages (reference category: GOLD stage 1).

GOLD ^a^		B	S.E.	Wald	df	Sig.	Exp (B)	95% CI for Exp (B)	
								Lower Bound	Upper Bound
2	Intercept	−2.906	3.224	0.812	1	0.367			
	Age	0.005	0.037	0.019	1	0.891	1.005	0.935	1.081
	Pack-year index	0.143	0.062	5.347	1	0.021	1.154	1.022	1.303
	mMRC	3.807	1.406	7.334	1	0.007	45.006	2.862	707.626
	CAT	−0.087	0.044	3.847	1	0.050	0.917	0.840	1.000
	FEV1	−0.018	0.017	1.119	1	0.290	0.982	0.950	1.015
	Sex = 1	0.018	0.831	0.000	1	0.983	1.018	0.200	5.187
	Smoker	−2.625	1.881	1.947	1	0.163	0.072	0.002	2.893
	Oxygen therapy	−0.584	0.861	0.460	1	0.498	0.558	0.103	3.014
3	Intercept	−5.376	3.823	1.977	1	0.160			
	Age	−0.045	0.046	0.979	1	0.322	0.956	0.874	1.045
	Pack-year index	0.155	0.064	5.900	1	0.015	1.167	1.030	1.322
	mMRC	7.100	1.502	22.333	1	<0.001	1212.323	63.784	23,042.201
	CAT	−0.141	0.061	5.283	1	0.022	0.868	0.770	0.979
	FEV1	−0.020	0.020	0.988	1	0.320	0.980	0.942	1.020
	Sex = 1	−0.373	1.015	0.135	1	0.713	0.689	0.094	5.031
	Smoker	−2.599	2.487	1.092	1	0.296	0.074	0.001	9.728
	Oxygen therapy	−1.126	0.995	1.283	1	0.257	0.324	0.046	2.278
4	Intercept	−11.513	4.184	7.570	1	0.006			
	Age	−0.083	0.050	2.811	1	0.094	0.920	0.835	1.014
	Pack-year index	0.200	0.064	9.645	1	0.002	1.222	1.077	1.386
	mMRC	9.555	1.565	37.273	1	<0.001	14,114.418	656.880	303,277.257
	CAT	−0.202	0.069	8.544	1	0.003	0.817	0.714	0.936
	FEV1	−0.019	0.022	0.797	1	0.372	0.981	0.940	1.023
	Sex = 1	−0.470	1.107	0.180	1	0.671	0.625	0.071	5.474
	Smoker	−3.435	2.641	1.691	1	0.193	0.032	0.000	5.708
	Oxygen therapy	−1.361	1.056	1.661	1	0.198	0.256	0.032	2.032

^a^. The reference category is 1.

**Table 4:** Binary logistic regression analysis of factors associated with GOLD stage 4 COPD (reference category: GOLD stages 1–3).

		B	S.E.	Wald	df	Sig.	Exp (B)	95% CI for EXP (B)	
								Lower	Upper
Step 1 ^a^	Age	−0.043	0.022	3.898	1	0.048	0.958	0.918	1.000
	Sex (1)	0.114	0.485	0.055	1	0.814	1.121	0.433	2.901
	Pack-year index	0.046	0.011	16.500	1	<0.001	1.047	1.024	1.071
	Smoker (1)	0.864	0.992	0.759	1	0.384	2.373	0.340	16.579
	mMRC	2.685	0.421	40.737	1	<0.001	14.657	6.426	33.427
	CAT	−0.064	0.035	3.338	1	0.068	0.938	0.876	1.005
	FEV1	0.000	0.009	0.001	1	0.982	1.000	0.982	1.018
	Oxygen therapy (1)	0.244	0.395	0.380	1	0.537	1.276	0.588	2.768
	Constant	−7.799	2.235	12.171	1	<0.001	0.000		

^a^. Variable(s) entered in step 1: age, sex, pack-year index, smoker, mMRC, CAT, FEV1, oxygen therapy.

**Table 5:** Binary logistic regression analysis of factors associated with clinically significant psychological distress (DASS depression ≥ 2).

DASS—Depression									
		B	S.E.	Wald	df	Sig.	Exp (B)	95% CI for EXP (B)	
								Lower	Upper
Step 1 ^a^	GOLD binary (1)	−0.046	0.358	0.016	1	0.899	0.955	0.473	1.929
	Age	−0.030	0.015	4.151	1	0.042	0.971	0.943	0.999
	Sex (1)	−0.212	0.309	0.473	1	0.492	0.809	0.442	1.481
	Pack-year index	−0.006	0.008	0.452	1	0.502	0.994	0.978	1.011
	Smoker (1)	−0.180	0.564	0.102	1	0.750	0.835	0.276	2.524
	mMRC	0.143	0.180	0.631	1	0.427	1.154	0.811	1.641
	CAT	0.125	0.024	27.096	1	<0.001	1.133	1.081	1.188
	FEV1	0.000	0.006	0.004	1	0.952	1.000	0.988	1.012
	Oxygen therapy (1)	0.311	0.270	1.326	1	0.250	1.364	0.804	2.314
	Constant	−0.809	1.311	0.381	1	0.537	0.445		

^a^. Variable(s) entered in step 1: GOLD binary, age, sex, pack-year index, smoker, mMRC, CAT, FEV1, oxygen therapy.

**Table 6:** Binary logistic regression analysis of factors associated with clinically significant anxiety symptoms (DASS anxiety ≥ 2).

DASS—Anxiety									
		B	S.E.	Wald	df	Sig.	Exp (B)	95% CI for EXP (B)	
								Lower	Upper
Step 1 ^a^	GOLD binary (1)	−0.294	0.398	0.545	1	0.460	0.745	0.342	1.626
	Age	−0.022	0.016	1.931	1	0.165	0.978	0.947	1.009
	Sex (1)	−0.196	0.340	0.331	1	0.565	0.822	0.422	1.602
	Pack-year index	0.006	0.009	0.391	1	0.532	1.006	0.988	1.023
	Smoker (1)	−0.804	0.740	1.181	1	0.277	0.447	0.105	1.909
	mMRC	−0.245	0.201	1.475	1	0.225	0.783	0.528	1.162
	CAT	0.165	0.026	39.291	1	<0.001	1.179	1.120	1.241
	FEV1	−0.003	0.007	0.228	1	0.633	0.997	0.984	1.010
	Oxygen therapy (1)	0.323	0.298	1.169	1	0.280	1.381	0.769	2.478
	Constant	−0.070	1.494	0.002	1	0.963	0.933		

^a^. Variable(s) entered in step 1: GOLD binary, age, sex, pack-year index, smoker, mMRC, CAT, FEV1, oxygen therapy.

**Table 7:** Binary logistic regression analysis of factors associated with clinically significant stress symptoms (DASS stress ≥ 2).

DASS—Stress									
		B	S.E.	Wald	df	Sig.	Exp (B)	95% CI for EXP (B)	
								Lower	Upper
Step 1 ^a^	GOLD binary (1)	0.075	0.378	0.039	1	0.843	1.078	0.514	2.261
	Age	−0.033	0.016	4.573	1	0.032	0.967	0.938	0.997
	Sex (1)	−0.255	0.321	0.629	1	0.428	0.775	0.413	1.455
	Pack-year index	0.004	0.009	0.186	1	0.666	1.004	0.987	1.021
	Smoker (1)	0.088	0.589	0.022	1	0.882	1.092	0.344	3.466
	mMRC	−0.081	0.187	0.189	1	0.664	0.922	0.639	1.330
	CAT	0.134	0.024	30.486	1	<0.001	1.144	1.090	1.199
	FEV1	−0.006	0.006	0.874	1	0.350	0.994	0.982	1.007
	Oxygen therapy (1)	0.384	0.284	1.828	1	0.176	1.468	0.841	2.563
	Constant	−0.184	1.370	0.018	1	0.893	0.832		

^a^. Variable(s) entered in step 1: GOLD binary, age, sex, pack-year index, smoker, mMRC, CAT, FEV1, oxygen therapy.

**Table 8:** Bayes factor independent sample test.

Bayes Factor Independent Sample Test (Method = Rouder) ^a^						
	Mean Difference	Pooled Std. Error Difference	Bayes Factor ^a^	t	df	Sig. (2-Tailed)
Clinic	−0.08	0.120	7.864	−0.658	283	0.511
Sex	−0.02	0.056	8.951	−0.407	283	0.684
Obesity	−0.04	0.064	8.105	−0.609	283	0.543
Smoker	0.05	0.032	2.459	1.685	283	0.093
Pack-year index	21.77	2.383	0.000	9.137	283	<0.001
Smoking cessation	0.03	0.048	7.722	0.686	283	0.493
mMRC	1.32	0.114	0.000	11.610	283	<0.001
Oxygen therapy	0.14	0.065	1.082	2.133	283	0.034
CAT	0.65	0.874	7.409	0.745	283	0.457
DASS-21—depression	0.03	0.154	9.520	0.198	283	0.843
DASS-21—anxiety	0.11	0.160	7.625	0.705	283	0.482
DASS-21—stress	0.06	0.138	8.857	0.433	283	0.665

^a^. Assumes unequal variance between groups.

**Table 9:** Descriptive statistics of clinical, respiratory, and psychological variables.

	Minimum	Maximum	Mean	Std. Deviation	Variance
Age	34	96	65.84	9	90
BP	0	1	0.72	0	0
FEV_1_	15.0	105.7	45.608	22	471
FVC	26.2	142.2	72.478	24	576
FEV1FVC	28.80%	94.00%	61.6681%	16%	261
Pack-year index	0	100	37.35	21	423
SpO_2_	52%	99%	92.68%	4%	18
mMRC	0	4	2.59	1	1
Oxygen therapy	0	1	0.46	0	0
CAT	7	37	23.44	7	44
MARS	0	1	0.72	0	0
WHO-5	20%	92%	50.76%	19%	343
DASS-21—depression	0	4	1.79	1	1
DASS-21—anxiety	0	4	1.87	1	1
DASS-21—stress	0	4	1.75	1	1
Valid N (listwise)					

BP—blood pressure; FEV_1_—forced expiratory volume in one second; FVC—forced vital capacity; SpO_2_—peripheral oxygen saturation; MARS—Medication Adherence Report Scale; WHO-5—World Health Organization–Five Well-Being Index.

**Table 10:** Descriptive statistics of DASS-21 depression, anxiety, and stress scores across GOLD stages.

			N	Mean	Std. Deviation	S.E.	95% CI for Mean	
							Lower Bound	Upper Bound
DASS-21 Depression	1		29	2.10	1.345	0.250	1.59	2.62
	2		69	1.48	1.313	0.158	1.16	1.79
	3		106	1.90	1.041	0.101	1.70	2.10
	4		81	1.81	1.108	0.123	1.57	2.06
	Total		285	1.79	1.173	0.069	1.66	1.93
	Model	Fixed Effects			1.163	0.069	1.66	1.93
		Random Effects				0.120	1.41	2.17
DASS-21 Anxiety	1		29	2.24	1.244	0.231	1.77	2.71
	2		69	1.55	1.388	0.167	1.22	1.88
	3		106	1.92	1.096	0.106	1.70	2.13
	4		81	1.95	1.150	0.128	1.70	2.20
	Total		285	1.87	1.213	0.072	1.73	2.01
	Model	Fixed Effects			1.203	0.071	1.73	2.01
		Random Effects				0.125	1.47	2.27
DASS-21 Stress	1		29	1.83	1.104	0.205	1.41	2.25
	2		69	1.36	1.124	0.135	1.09	1.63
	3		106	1.94	0.934	0.091	1.76	2.12
	4		81	1.79	1.033	0.115	1.56	2.02
	Total		285	1.75	1.048	0.062	1.63	1.87
	Model	Fixed Effects			1.028	0.061	1.63	1.87
		Random Effects				0.141	1.30	2.20

**Table 11:** ANOVA effect size estimates for DASS-21 across GOLD stages.

ANOVA Effect Sizes ^a,b^				
		Point Estimate	95% CI	
			Lower	Upper
DASS-21—Depression	Eta-squared	0.028	0.000	0.067
	Epsilon-squared	0.017	−0.011	0.057
	Omega-squared fixed-effect	0.017	−0.011	0.057
	Omega-squared random-effect	0.006	−0.004	0.020
DASS-21—Anxiety	Eta-squared	0.028	0.000	0.068
	Epsilon-squared	0.018	−0.011	0.058
	Omega-squared fixed-effect	0.018	−0.011	0.057
	Omega-squared random-effect	0.006	−0.004	0.020
DASS-21—Stress	Eta-squared	0.047	0.006	0.095
	Epsilon-squared	0.037	−0.005	0.086
	Omega-squared fixed-effect	0.037	−0.005	0.085
	Omega-squared random-effect	0.013	−0.002	0.030

^a^. Eta-squared and epsilon-squared are estimated based on the fixed-effect model. ^b^. Negative but less biased estimates are retained, not rounded to zero.

## Data Availability

The original contributions presented in this study are included in the article/Supplementary Material. Further inquiries can be directed to the corresponding author.

## Supplementary Materials

The following supporting information can be downloaded at: https://www.mdpi.com/article/10.3390/medsci14010147/s1, Figures S1–S9 (Bayesian posterior distribution plots for independent sample comparisons), Table S1 (population characteristics according to GOLD stage), and Tables S2 and S3 (Levene’s test for homogeneity of variances and full one-way ANOVA results).

## Abbreviations

ANOVA	Analysis of variance
aOR	Adjusted odds ratio
BF_01_	Bayes factor in favor of the null hypothesis relative to the alternative
BP	Blood pressure
CAT	COPD Assessment Test
CI	Confidence interval
COPD	Chronic obstructive pulmonary disease
DAG	Directed acyclic graph
DASS-21	Depression Anxiety Stress Scales–21
EPV	Events per variable
FEV_1_	Forced expiratory volume in one second
FVC	Forced vital capacity
FEV_1_/FVC	Ratio of forced expiratory volume in one second to forced vital capacity
GOLD	Global Initiative for Chronic Obstructive Lung Disease
mMRC	Modified Medical Research Council dyspnea scale
MARS	Medication Adherence Report Scale
OR	Odds ratio
SpO_2_	Peripheral oxygen saturation
VIF	Variance inflation factor
WHO-5	World Health Organization–Five Well-Being Index
