# Supplementary material for: Psychological Distress in COPD Assessed by DASS-21-R: Multivariable Regression and Bayesian Analysis Across GOLD Stages
Source: Med Sci (Basel). 2026 Mar 19;14(1):147. doi: 10.3390/medsci14010147 (PMC13028331; doi:10.3390/medsci14010147)
Supplement: Supplementary file 1 [file medsci-14-00147-s001.zip › medsci-4145733-supplementary.pdf]

SUPPLEMENTARY MATERIAL

Table S1. Population characteristics according to GOLD severity

	GOLD	N	Mean	Std. Deviation	Std. Error Mean
Clinic	< 4	204	2.05	.911	.064
	= 4	81	1.98	.908	.101
Sex	< 4	204	1.25	.431	.030
	= 4	81	1.22	.418	.046
Obesity	< 4	204	.40	.490	.034
	= 4	81	.36	.482	.054
Smoker	< 4	204	.92	.270	.019
	= 4	81	.98	.156	.017
Packs years index	< 4	204	31.17	19.859	1.390
	= 4	81	52.94	12.800	1.422
Smoking cessation	< 4	204	.15	.360	.025
	= 4	81	.19	.391	.043
mMRC	< 4	204	2.21	.972	.068
	= 4	81	3.53	.502	.056
Oxygen therapy	< 4	204	.42	.494	.035
	= 4	81	.56	.500	.056
CAT	< 4	204	23.25	6.940	.486
	= 4	81	23.90	5.858	.651
DASS 21 - Depression	< 4	204	1.78	1.200	.084
	= 4	81	1.81	1.108	.123
DASS 21 - Anxiety	< 4	204	1.84	1.239	.087
	= 4	81	1.95	1.150	.128
DASS 21 - Stress	< 4	204	1.73	1.056	.074
	= 4	81	1.79	1.033	.115

Figure S1: Bayesian estimation of the mean difference in clinic variable between GOLD <4 and GOLD =4 groups

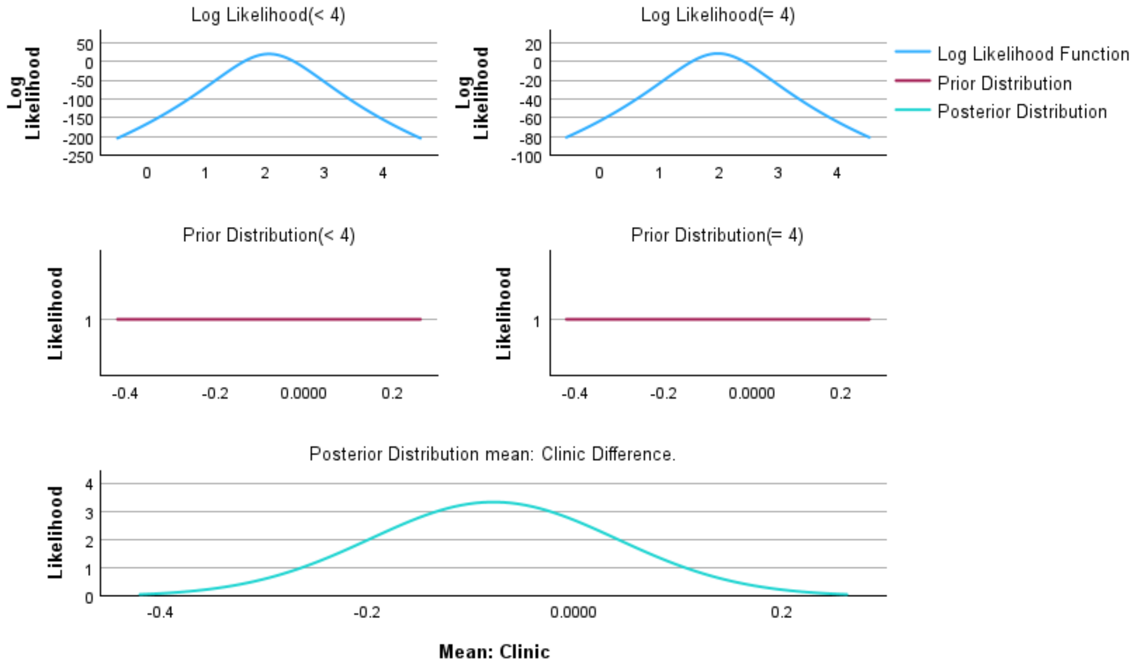

Figure S2. Bayesian estimation of the mean difference in sex between GOLD <4 and GOLD =4 groups

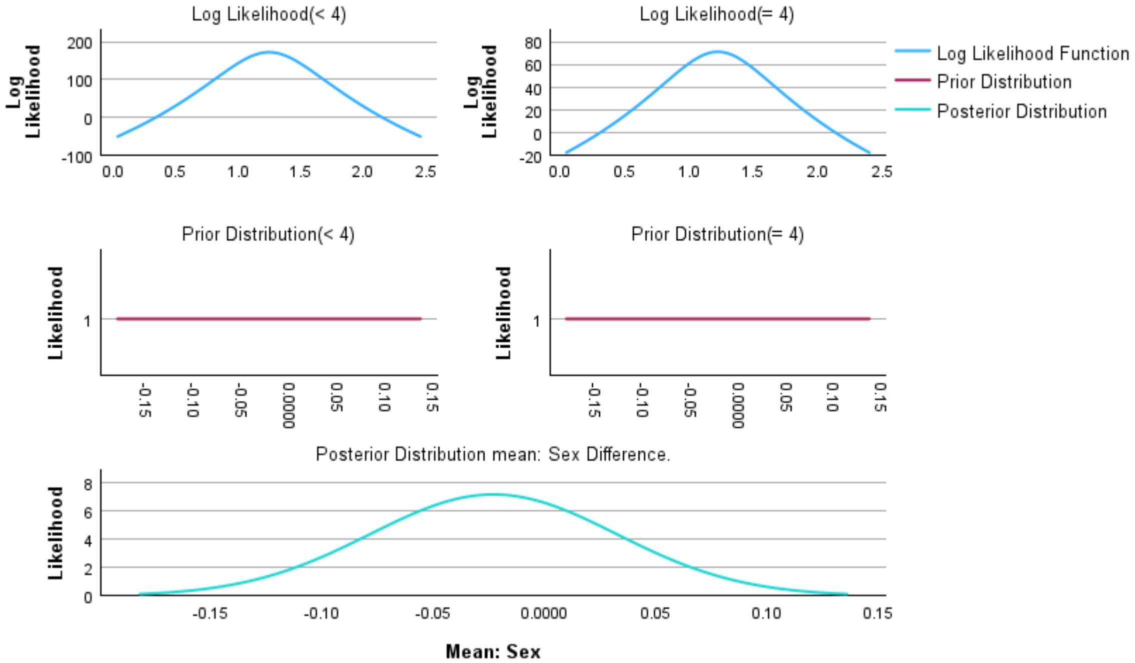

Figure S3. Bayesian estimation of the mean difference in obesity between GOLD <4 and GOLD =4 groups

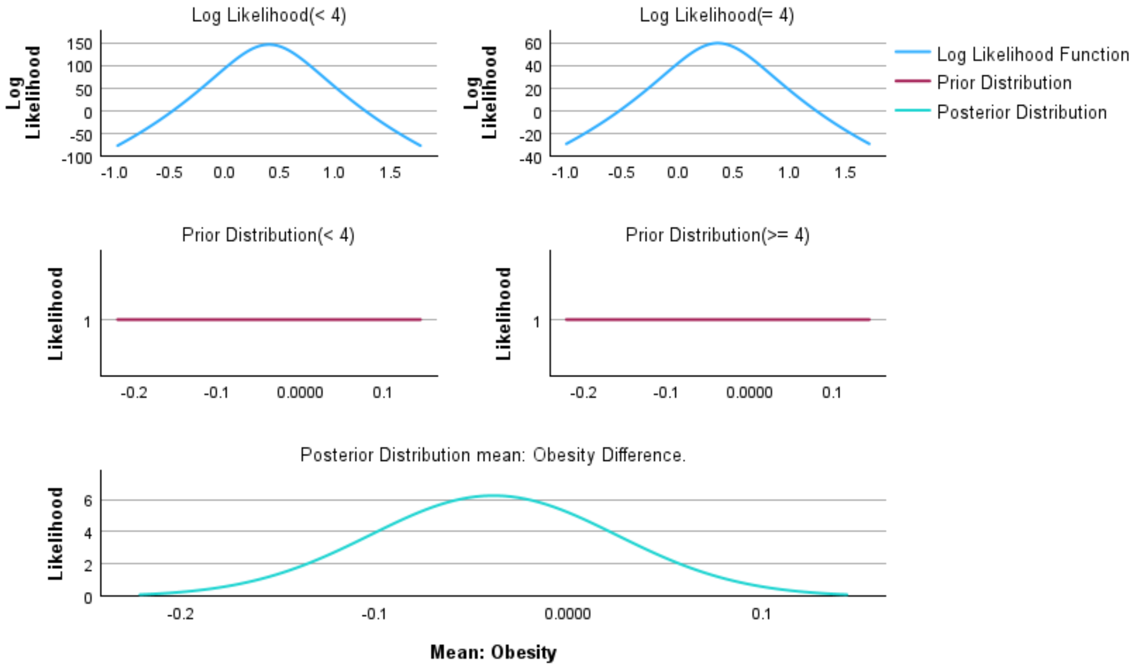

Figure S4. Bayesian estimation of the mean difference in smokers between GOLD <4 and GOLD =4 groups

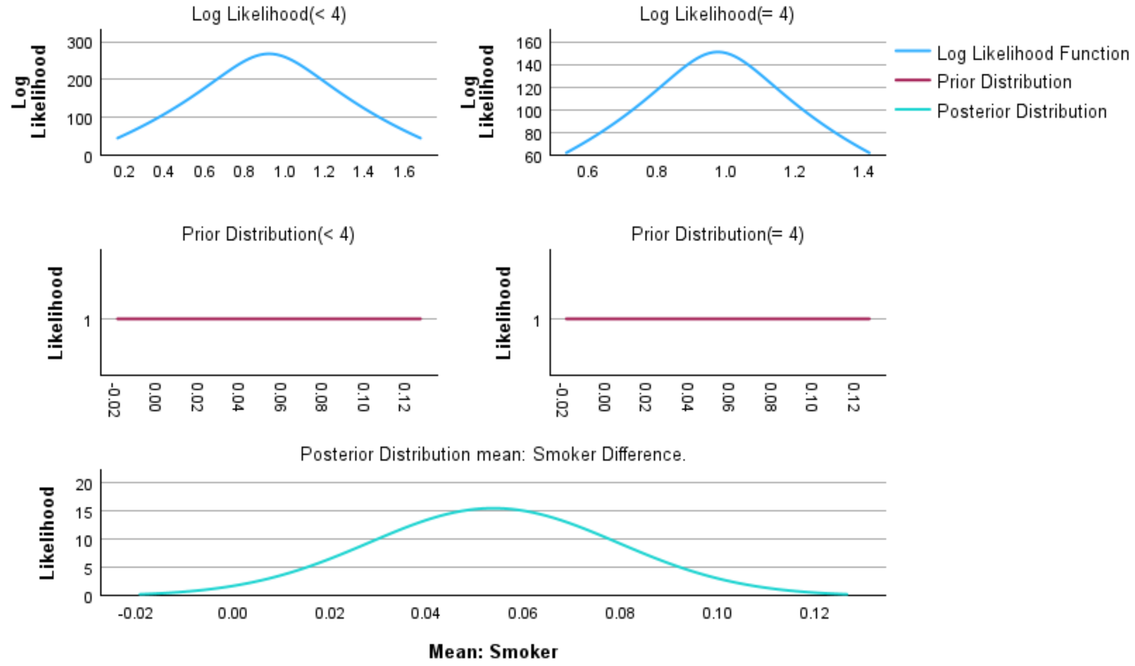

Figure S5. Bayesian estimation of the mean difference in pack-year index between GOLD <4 and GOLD =4 groups

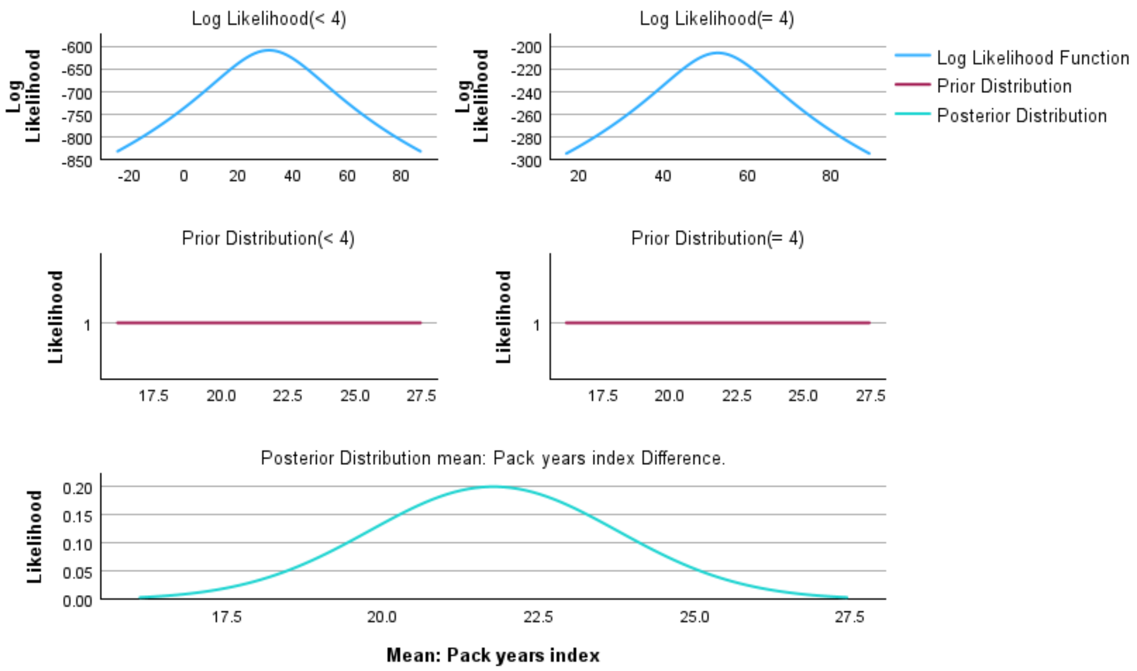

Figure S6. Bayesian estimation of the mean difference in smoking ceasation between GOLD <4 and GOLD =4 groups

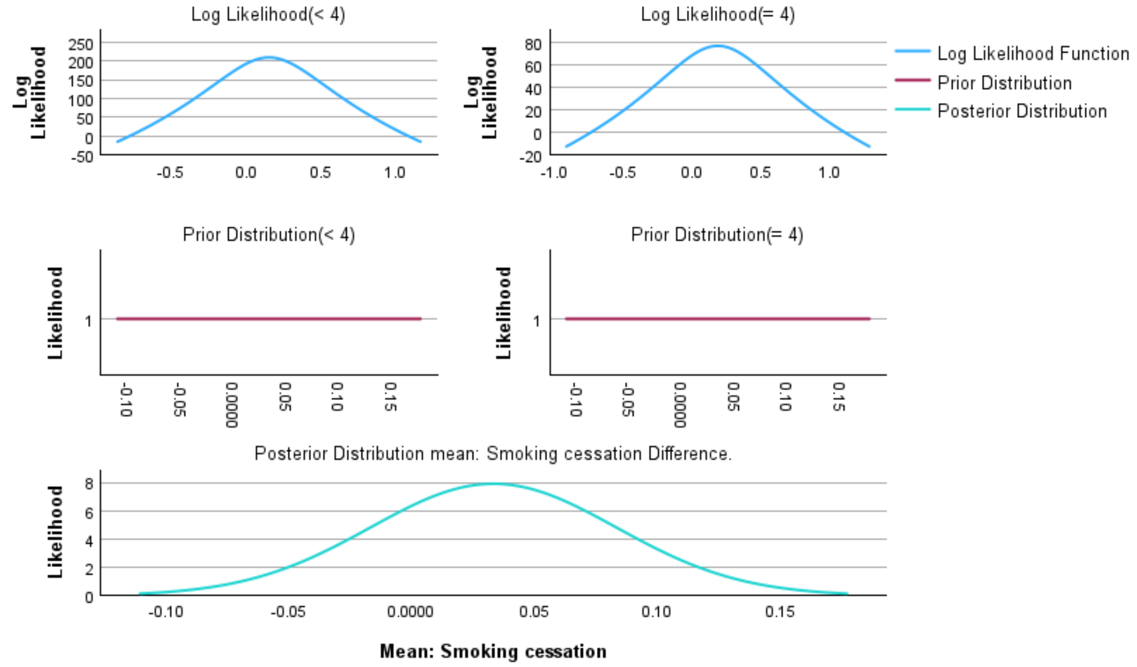

Figure S7. Bayesian estimation of the mean difference in sex between GOLD <4 and GOLD =4 groups

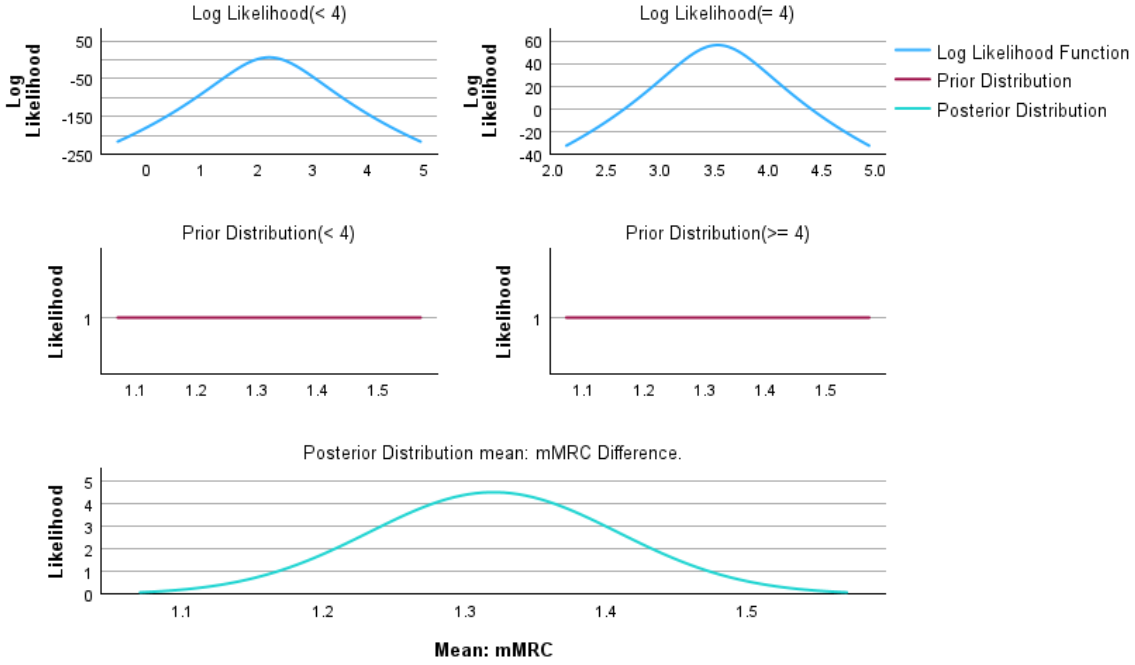

Figure S8. Bayesian estimation of the mean difference in Oxygen therapy between GOLD <4 and GOLD =4 groups

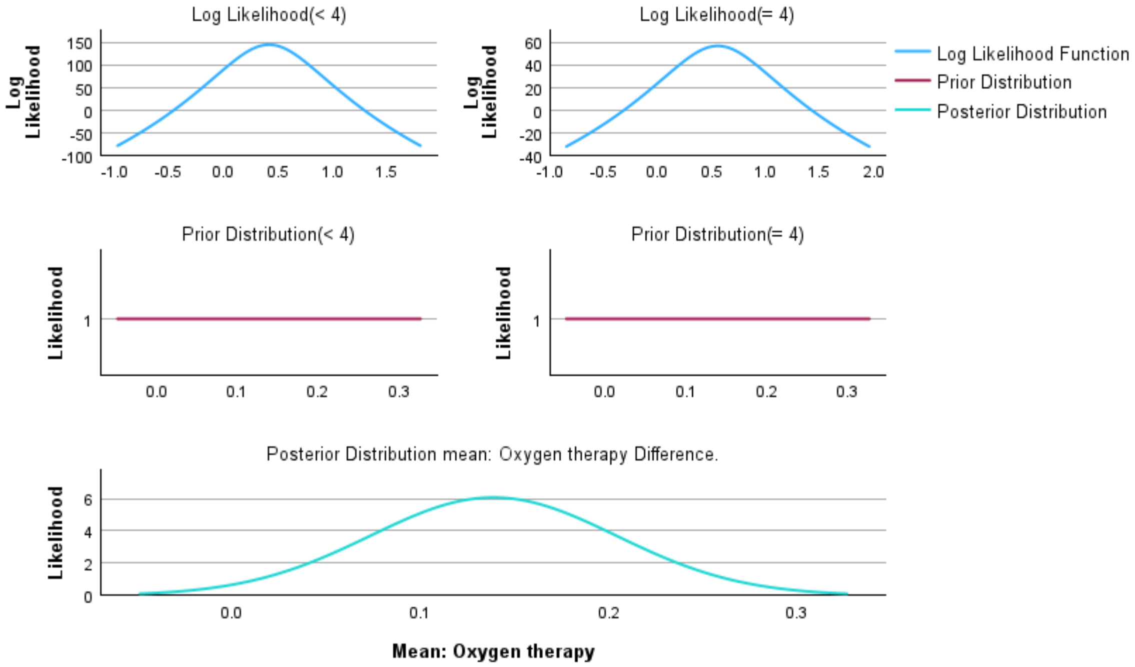

Figure S9. Bayesian estimation of the mean difference in CAT between GOLD <4 and GOLD =4 groups

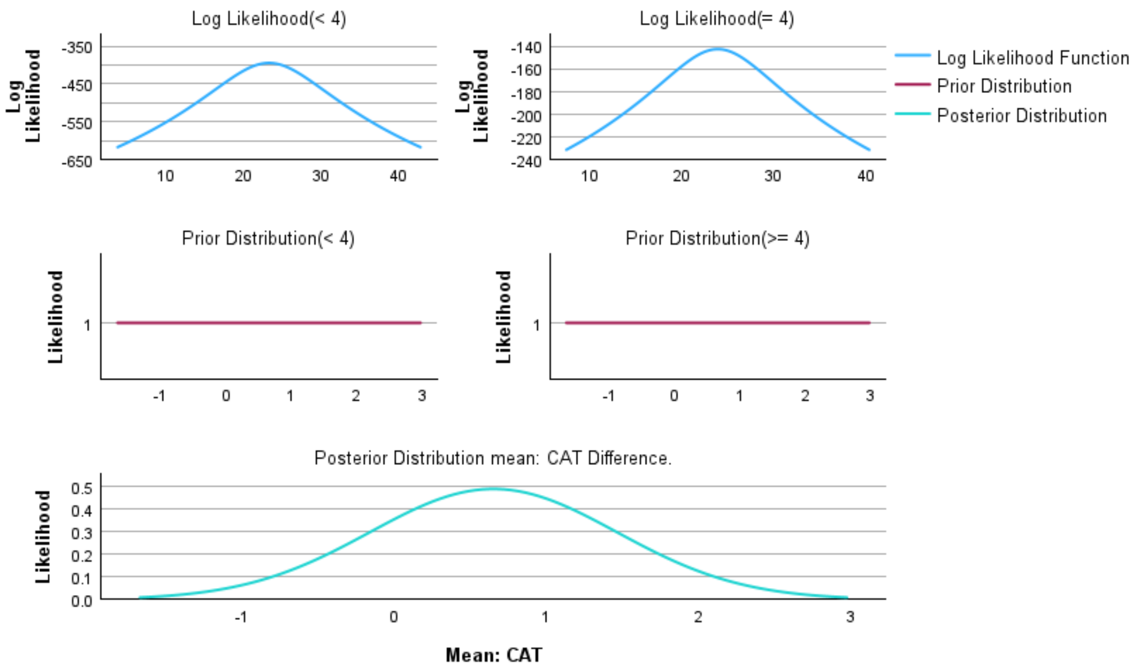

ANOVA analysis

Table S2. Tests of Homogeneity of Variances

		Levene Statistic	df1	df2	Sig.
DASS 21 - Depression	Based on Mean	3.548	3	281	.015
	Based on Median	1.991	3	281	.116
	Based on Median and with adjusted df	1.991	3	252.342	.116
	Based on trimmed mean	3.321	3	281	.020
DASS 21 - Anxiety	Based on Mean	4.250	3	281	.006
	Based on Median	3.403	3	281	.018
	Based on Median and with adjusted df	3.403	3	277.419	.018
	Based on trimmed mean	4.322	3	281	.005
DASS 21 -Stress	Based on Mean	4.487	3	281	.004
	Based on Median	3.721	3	281	.012

	Based on Median and with adjusted df	3.721	3	278.246	.012
	Based on trimmed mean	4.601	3	281	.004

Table S3. One-way ANOVA analysis of DASS-21 domains across GOLD stages

		Sum of Squares	df	Mean Square	F	Sig.
DASS 21 - Depression	Between Groups	10.798	3	3.599	2.662	.048
	Within Groups	379.988	281	1.352		
	Total	390.786	284			
DASS 21 - Anxiety	Between Groups	11.775	3	3.925	2.714	.045
	Within Groups	406.421	281	1.446		
	Total	418.196	284			
DASS 21 - Stress	Between Groups	14.638	3	4.879	4.614	.004
	Within Groups	297.172	281	1.058		
	Total	311.811	284			
